# Ten simple rules for building a collaborative coding culture

**DOI:** 10.1371/journal.pcbi.1013970

**Published:** 2026-02-23

**Authors:** Austin L. Zuckerman, Sarah Faber, Kelly Shen, Anthony R. McIntosh, Ashley L. Juavinett

**Affiliations:** 1 Program in Mathematics and Science Education, University of California San Diego, La Jolla, California, United States of America; 2 Program in Mathematics and Science Education, San Diego State University, San Diego, California, United States of America; 3 Institute for Neuroscience and Neurotechnology, Simon Fraser University, Burnaby, British Columbia, Canada; 4 Department of Biomedical Physiology and Kinesiology, Faculty of Science, Simon Fraser University, Burnaby, British Columbia, Canada; 5 Neurobiology Department, School of Biological Sciences, University of California San Diego, La Jolla, California, United States of America; Dassault Systemes BIOVIA, UNITED STATES OF AMERICA

## Overview

Programming is quickly becoming an integral part of many biology labs. However, with limited formal training of their own, few researchers know how to onboard new programmers and encourage the growth of computing skills. Here, we integrate observations from our own experiences with interviews of more than 25 researchers across career stages to identify barriers and supports to developing programming skills in the lab. We then turn these insights into ten concrete recommendations for research group leaders, especially those who conduct research without a computational focus. These recommendations include holding coding office hours, code review processes, and discussions around generative AI. Ultimately, we believe that such practices can empower labs to produce more accessible, efficient, and reproducible research code bases.

## Introduction

With the growth in size and complexity of datasets, models, and the questions we hope to address with them, computer programming is a necessary skill for researchers across subfields of biology. Yet, there is limited guidance on how to encourage computational skill building either informally or formally, or how to create an effective and collaborative coding culture within a lab. This article aims to address this gap.

Some early career researchers learn programming skills via coursework, but historically, most biologists (especially those without a computational focus) have obtained these skills via self-guided learning, driven by the idea that it is a “necessary evil.” The effectiveness of such self-guided learning is variable; while there are abundant and accessible resources for self-guided learning and many card-carrying computer scientists are self-taught, these resources are often not structured in terms of allocated time and pedagogical scaffolding and are not tailored to the needs of a specific field of research. Further, our previous work on an undergraduate programming course in biology has illustrated that those who are self-taught may not view their own skill building as authentic and may thereby have stronger feelings of being a computational imposter [1]. Finally, there are notable risks to reproducibility when lab members learn programming individually and the lab’s code lives tucked away on personal computers. Indeed, other researchers have noted the threats to reproducibility and scientific integrity in code that is written by learners without appropriate collaborative support [2–4].

On the other hand, there are clear benefits to a collaborative coding environment and a culture for learning: all lab members, not just those with predispositions towards programming, can acquire the proper foundation to read, edit, and accurately write their own code. Although artificial intelligence (AI) assistants show promise for debugging and simplifying code, using them to write code *de novo* without any prior programming framework is problematic. Instead, creating a culture and set of shared practices for learning programming, including conversations about whether to use AI, can help quickly upskill new lab members and empower labs to scale their research beyond what is possible by tinkering with spreadsheets. This opens doors for career opportunities beyond the lab and creates sustainable and reproducible workflows for research.

Before we introduce our rules, it is important to frame the term “collaborative coding culture” from a critical lens so that readers are able to operationalize our rules appropriately. We acknowledge that computer science has a historical backdrop that is rife with gendered and racialized narratives, which have perpetuated problematic and limited perceptions of the kinds of people who can code [5]. To afford opportunities to *all* who want to learn computer programming, we must proactively counter this anti-pedagogical culture in our learning spaces. As researchers consider adopting the below rules to create a collaborative coding culture, we encourage them to critically evaluate how they put these results in practice by integrating inclusive principles such as avoiding “one size fits all” approaches that accommodate only the most “capable” trainees, acknowledging the stereotypes and individualistic framings that gatekeep minority groups out of programming spaces, and signaling a culture of mutual care and humility shared by trainers and trainees alike [6–7].

To develop these recommendations, semi-structured interviews were conducted with undergraduate researchers, graduate students, postdoctoral scholars, university faculty, and industry professionals who were recruited nationally using snowball sampling methods. These one-hour interviews were retrospective and invited participants to comprehensively reflect on their choices to pursue computational opportunities in biology, the learning and professionalization experiences that were influential in orienting them to pursue computational work, as well as the types of support and resources that affected their experiences in these pathways. These interviews are part of a broader study examining decisions to persist in computing pathways in biology, and the comments used to illustrate the recommendations below emerged from a thematic analysis using an inductive coding approach.

**Fig 1:**
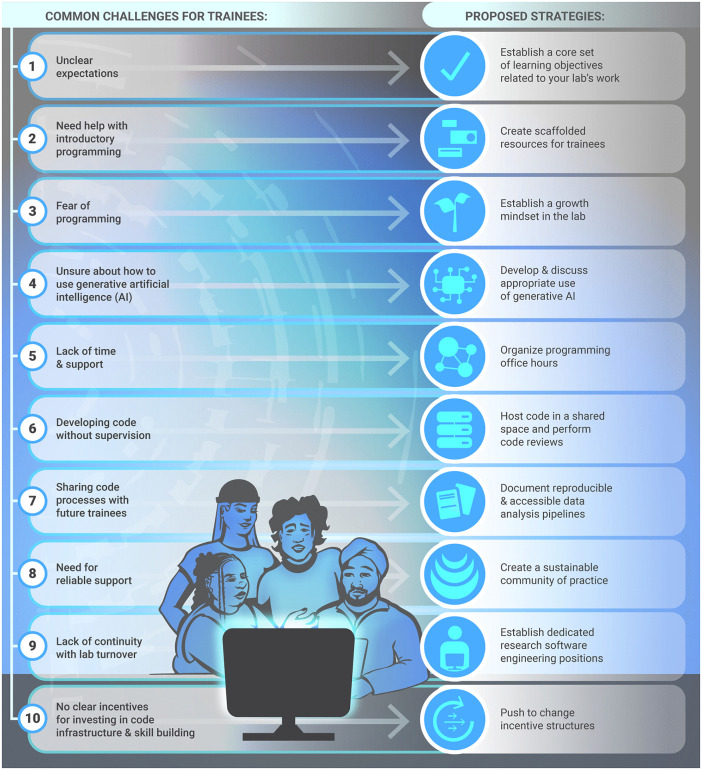
Common challenges in learning programming for research trainees, along with proposed strategies to address them.

Drawing from our own experience and these qualitative interviews, here we motivate the need for more structured programming skill building for current researchers and provide concrete advice for creating cultures of learning within research labs (see Fig 1 for an overview).

## Creating the foundation

First things first, it is important to explicitly describe how programming is used in your lab and what expectations you have for trainees on particular projects. Then, you can begin to develop a common set of resources and a community of practice for programming in your lab.

### Rule 1. Establish programming learning objectives

A strong foundation for programming skill development requires determining a common minimum skill set that *all* trainees can learn. For example, work in your lab could require proficiency in Python, such as using custom scripts to read and visualize neural recording time series. Alternatively, trainees may need to be able to organize file structures and run command-line genomics analyses. Effective learning objectives are specific and measurable (e.g., “Generate clear and well-labeled figures describing participants’ task performance.”). Although not every learning objective will apply to every project (or every trainee), some attention to what a core set of objectives *might* be is a worthwhile challenge. Once a minimum skill set is defined, trainees can learn these skills through a combination of formal and informal learning opportunities, creating a framework that allows trainees to progressively build their skills while ensuring that foundational tools and techniques are shared across different levels of expertise.

### Rule 2. Identify *necessary* uses of programming

A related challenge for trainees and their mentors is identifying under *what contexts* programming should be used in the analysis pipeline. For example, trainees may spend countless hours annotating figures programmatically when they could have just as readily customized their figures using GUI tools (e.g., vector-based graphic design programs). While learning to code is increasingly normalized as an essential skill in contemporary research, trainees may also need to be explicitly reminded that it is *not the only tool* that should be relied upon for every aspect of the analysis pipeline. The opposing needs here are reproducibility and ease of use: creating figures programmatically easily allows for reproduction, but can be tedious for a novice programmer. Trainees should be encouraged to generate individual plots in a reproducible manner, which typically means programmatically, but can compile plots into composite figures and add other annotations and panel labels in a GUI. Even still, well-documented and shared spreadsheets can be appropriate and reasonably reproducible tools. Creating a set of guidelines that helps identify situations where automation or computational analysis is essential (e.g., handling large datasets or applying complex statistical methods) versus when GUI tools are better suited can help strike a balance so that these tools can work in tandem rather than as competing solutions.

### Rule 3. Establish a common set of resources

Establishing a common foundation through a centralized and scaffolded set of resources can ensure that all trainees learn fundamentals and then build upon this foundation as needed for their specific research projects. Curating detailed notebooks and/or ReadMe and Wiki pages for particular analysis pipelines—including step-by-step code, explanations of key analysis decisions, and annotated examples—should be treated as equally important as establishing protocols for experimental reproducibility [8,9]. For example, the McIntosh lab has created its own GitHub organization with a mix of public and private repositories of analysis pipelines, tools for common tasks like data extraction and output visualization, and a collection of training and usage resources (https://github.com/McIntosh-Lab). Building on this, labs should also consider adopting standardized directory structures and pre-configured programming environments for ease and reproducibility (for more details and ideas, see [10–13]).

### Rule 4. Establish standards for ethical and effective use of AI tools

There are numerous commonly-used models under the AI umbrella in the sciences (deep neural nets, Bayesian discovery, etc.), but this section will specifically address the recent proliferation of large language models (LLMs) in generating, evaluating, and explaining research code.

The emergence of generative AI tools presents both challenges and opportunities for programming education in biology. The authors of this manuscript were divided on the use of generative AI in programming education, and we present both viewpoints here.

We do not condone uncritical adoption of AI, but several authors contend that it *can* be helpful for learning as well as revising or debugging drafts of code, as others have noted [14,15]. Rather than avoiding these tools, labs could actively discuss best practices for their integration, such as for streamlining certain aspects of data analysis (e.g., exploratory analyses), explaining existing lab code bases for new users, or generating programmatic backbones for additional analyses. These discussions should include determining when AI assistance is appropriate, how to verify AI-generated code with unit tests, and how to balance AI use with fundamental skill development. Importantly, any AI generated code needs to be carefully verified by lab members with programming experience.

On the other hand, other authors of this manuscript are concerned about *any* use of LLMs. While efforts are being made to provide guidelines for LLM usage for programming, there are numerous outstanding issues related to ethics, replicability, and accountability [16]. Code generated with LLMs may be inaccurate (especially for custom applications) and introduces the risk of researchers unable to describe, change, or troubleshoot the computational backbone of their experiments or analyses, which presents a very real threat to replicability and scientific integrity. Guest and colleagues detail the dangers of adopting LLMs uncritically, and the irony of supporting their use in places of learning [16]. Instead of offloading programming education to LLMs, researchers should challenge the prioritization of efficiency and individualism over supportive learning environments (as described in the next section). Science is inherently collaborative, and the stewardship of learning is the work of people. While advocates of AI use tout its usefulness in streamlining “onerous” processes like explaining existing lab code bases to new users, generating programmatic scaffolds for additional analyses, and reducing the time novice programmers spend seeking assistance from senior colleagues, these are valuable processes where true learning happens. If we seek to build a collaborative coding culture, it would be more productive to challenge the systems that view time spent on these learning processes as wasted effort rather than patching through them with LLMs and hoping for the best.

The inability to reach consensus on AI use within our small group of authors is likely representative of similar tensions within many research groups. We encourage labs to acknowledge these differences in viewpoints and maintain an ongoing open dialogue among their members to ultimately develop a shared framework that carefully weighs benefits and risks to AI adoption.

## Creating and maintaining a learning environment

Good science relies on good training, and good training relies on an environment that supports growth. Competent programmers don’t spring fully-formed from the ground; they are products of their environment. Creating and maintaining a supportive learning environment is vital to scientific training—in one researcher’s words, “I was lucky because I was in a lab where [coding] was a big focus and they put a lot of emphasis on people learning these skills well and using them correctly.” As with acquiring any skill, the initial knowledge gap can be intimidating, and this is where a good lab environment can make all the difference. But what makes a supportive environment?

### Rule 5. Normalize disclosing insecurities and asking questions

A supportive environment is one where learners have the freedom to fail without the fear of retribution or shame. Hypotheses fail, significance testing fails, results fail to replicate—science is full of failure! As such, a culture of learning and failing openly is beneficial to lab culture as well as learning new technical skills. This can take many forms and is best modeled “top-down” so new trainees understand that learning to program, like learning how to do research, is an iterative process. As one of our interview participants stated: “If we can normalize [for] wet lab that experiments will fail and things are hard, then I feel like we can also and we should also normalize that for computing. Not everyone is a superstar from the get-go. And it’s fine to learn.” Posting code snippets in lab-public spaces for review, devoting meeting time to code reviews [17], and including challenges, successes, and failures in these reviews rather than a “finished product” script or code base all contribute to a collaborative coding culture where everyone is able to share in learning (see [9–13] for additional tips and *The Good Research Code Handbook* (https://goodresearch.dev/) for a full helpful guide to writing good research code).

### Rule 6. Encourage collaboration around writing code

Another interview participant noted that “Helping somebody with their code is sometimes kind of like therapy. And I think that should be actually acknowledged.” Transparency and collaboration are not only important in our experimental design data collection but can and should also be baked into how we teach and learn programming. This can look like code reviews for projects in the lab, lab talks on code and analyses, and journal clubs focused on reviewing code from outside the lab. Hosting programming office hours where coding problems can be dissected and workshopped as a group is another strategy that fosters collaboration and promotes this culture of learning. Ultimately, inviting collaborators into coding tasks for scientific projects can also earn them authorship and recognition, something that could prove to be a fruitful positive feedback loop to incentivize and solidify research software engineering roles (see Rule 9 below).

Further, regular resource review and updating should be built into lab workflows rather than treated as optional side projects. For example, in the McIntosh lab, one hour a week is spent in “project management retreat”. These sessions provide dedicated time to hold colleagues accountable for performing data and code management tasks in an informal environment. This establishes the importance of project and code management in lab culture, ensures time is allocated to complete these tasks, and provides accountability for lab members at all levels of seniority.

### Rule 7. Develop an infrastructure for a collaborative code base

The maintenance of code bases and practices needs to be considered an essential part of a lab’s operations, akin to managing reagents or animal colonies. A common practice is to set up the lab as an organization on a code hosting site and then create individual repositories for specific projects. It is then important to ensure that multiple lab members have administrative privileges for these repositories, which provides necessary and beneficial redundancy and removes the burden from single lab members. As one former trainee we interviewed observed, “I spent the last six months of my PhD creating a programming manual for the lab, but I doubt anyone’s updated it since I left.”

### Rule 8. Establish an ongoing community of practice

The foundation for this culture is not complete without opportunities for trainees to participate in communities of practice that support their learning. A “community of practice” can be thought of as a group of individuals who collectively learn through shared goals and activities, gradually developing similar habits and ways of thinking [18,19]. As paraphrased from an interview participant, there is a need to “incentivize community-driven learning in that middle sphere where people need the right balance of trying something themselves and having their hand held a little bit.” Dedicated time within research for participating in learning communities is essential for trainees to engage meaningfully without the competing pressures of other responsibilities. These communities could include multi-week departmental workshops that engage multiple labs (hosted by a department or broader organization, e.g., Neuromatch) and create shared learning environments, as well as formalized computer programming curricula integrated into graduate programs (see [20] for a concrete example of a three-stage working model for such a community, and [21] for a similar example for a computational biology community).

## Systemic change

The most significant challenge in maintaining programming learning environments is sustainability. While individual labs may develop excellent resources and practices, these often deteriorate when key personnel depart. As such, there are several broader principles for systemic, sustainable change that labs could consider, outlined below.

### Rule 9. Establish dedicated research software engineering positions

Improving sustainability for programming practices requires both lab-level practices (like those described above) and institutional support. At the institutional level, dedicated positions for programming specialists within departments can provide stable support across lab transitions. For example, positions such as “Research Software Engineer” or “Computational Methods Specialist” could be created, where the person maintains central resources, conducts workshops, and provides consistent support across research groups [22,23]. Such positions acknowledge that programming education requires dedicated time and expertise that individual PIs may not possess or have the bandwidth to provide.

The position should also liaise with the university IT cores, providing an opportunity to ensure code development is done while being mindful of the infrastructure to support code execution (e.g., dedicated workstations, HPC clusters). This opens the potential for discipline-specific coding groups that may be a mix of support staff and lab personnel, setting up an environment to better support coding needs with the added benefit of providing training and growth opportunities for each team as they amass critical skills and become more proficient at supporting code development—a skill valued beyond academia.

### Rule 10. Push to change incentive structures

The sustainability question ultimately relies on incentive structures within academia and recognizing data and software contributions in hiring and promotion. Fortunately, this is a growing trend: recent study in the United Kingdom found that a third of academic institutional repositories, which showcase scholarly artifacts from their researchers, recognize software as scholarly work [24]. Until programming education and tool development are recognized and rewarded via scholarly artifacts (e.g., DOIs) and in tenure and promotion decisions, these crucial activities will continue to be undervalued (see [25] for ten simple rules for doing so). While some institutions have begun acknowledging software packages and educational resources in evaluation processes, broader systemic change is needed to ensure that those creating these resources receive appropriate recognition [26]. Institutionalizing our recognition of software development work in the lab and building inclusive cultures for learning programming can bring more scientists into computing and ultimately improve the kinds of science we do.
